# AMPA Receptor Activation Promotes Non-Amyloidogenic Amyloid Precursor Protein Processing and Suppresses Neuronal Amyloid-β Production

**DOI:** 10.1371/journal.pone.0078155

**Published:** 2013-10-24

**Authors:** Sarah E. Hoey, Federica Buonocore, Carla J. Cox, Victoria J. Hammond, Michael S. Perkinton, Robert J. Williams

**Affiliations:** 1 King's College London, Wolfson Centre for Age-Related Diseases, London, United Kingdom; 2 University of Bath, Department of Biology and Biochemistry, Bath, United Kingdom; University of Melbourne, Australia

## Abstract

Soluble oligomeric amyloid β peptide (Aβ) generated from processing of the amyloid precursor protein (APP) plays a central role in the pathogenesis of Alzheimer's Disease (AD) and through actions at glutamatergic synapses affects excitability and plasticity. The physiological control of APP processing is not fully understood but stimulation of synaptic NMDA receptors (NMDAR) can suppress Aβ levels through an ERK-dependent increase in α-secretase activity. AMPA-type glutamate receptors (AMPAR) couple to ERK phosphorylation independently of NMDAR activation raising the possibility that stimulation of AMPAR might similarly promote non-amyloidogenic APP processing. We have tested this hypothesis by investigating whether AMPAR directly regulate APP processing in cultured mouse cortical neurons, by analyzing APP C-terminal fragments (CTFs), soluble APP (sAPP), Aβ levels, and cleavage of an APP-GAL4 reporter protein. We report that direct stimulation of AMPAR increases non-amyloidogenic α-secretase-mediated APP processing and inhibits Aβ production. Processing was blocked by the matrix metalloproteinase inhibitor TAPI-1 but was only partially dependent on Ca^2+^ influx and ERK activity. AMPAR can therefore, be added to the repertoire of receptors that couple to non-amyloidogenic APP processing at glutamatergic synapses and thus pharmacological targeting of AMPAR could potentially influence the development and progression of Aβ pathology in AD.

## Introduction

Generation of the amyloid β peptide (Aβ) from the amyloid precursor protein (APP) and the subsequent aggregation of Aβ as soluble synaptotoxic oligomers, is central to the pathogenesis of Alzheimer's Disease (AD) [1]–[4]. The production of Aβ via the amyloidogenic pathway of APP processing begins with β-secretase cleavage followed by γ-secretase cleavage to yield sAPPβ and Aβ [5]. Alternatively APP can be cleaved by α-secretase followed by γ-secretase to yield a p3 fragment and neurotrophic sAPPα in a non-amyloidogenic pathway [6]. The identification of new approaches for reducing Aβ burden either by inhibition of βγ -secretases, stimulation of α-secretase, enhanced Aβ clearance, or by maintaining neuronal homeostasis remains a major therapeutic goal for AD, particularly in the very early stages of pathogenesis. The mechanisms that regulate APP processing under pathological conditions have been extensively studied, but less is known about the physiological control of endogenous non-mutated APP processing at healthy, non-diseased synapses although there is a close relationship between APP processing, Aβ production and the regulation of excitability at glutamatergic synapses [7]–[9]. Synaptic activity enhances Aβ release from nerve terminals following endocytosis of APP into endosomes [10], [11] and similarly stimulation of presynaptic group II metabotropic glutamate receptors (mGluRs) increases Aβ secretion [12]. In contrast, postsynaptic group I mGluRs promote non-amyloidogenic α-secretase mediated APP processing [13]. However, the situation with respect to N-Methyl-D-Aspartate receptors (NMDAR) is more complex, as stimulation of NMDAR can either elevate or inhibit Aβ production depending upon temporal and spatial differences in receptor-evoked signaling events. For example, prolonged activation of NMDAR with sub-maximal doses of agonist [14], [15] or specific stimulation of extrasynaptic NMDAR [16], promotes amyloidogenic processing of APP and hence increases Aβ production. In contrast, direct activation of synaptic NMDAR favours non-amyloidogenic α-secretase-mediated APP processing to reduce Aβ production and release [17], first recruiting ADAM-10, towards the cell surface [18] and then upregulating ADAM-10 expression in an ERK-dependent manner [19]. Administration of NMDAR antagonists or channel blockers increases Aβ levels both *in vitro* and *in vivo* [15], [17] and similar elevations in Aβ are observed following the administration of MEK/ERK inhibitors [15]. Thus, basal activity at NMDAR suppresses Aβ levels through a potentially ERK-dependent increase in α-secretase activity suggesting that other receptors found at glutamatergic synapses that act via ERK might similarly suppress Aβ production.

α-amino-3-hydroxy-5-methyl-4-isoxazolepropionic acid (AMPA)-type glutamate receptors (AMPAR) couple to ERK phosphorylation independently of NMDAR activation [20], [21], raising the possibility that stimulation of AMPAR might lead to promotion of non-amyloidogenic APP processing. We have tested this hypothesis by investigating whether AMPAR activity directly regulates APP processing in cultured mouse cortical neurons, by analyzing APP C-terminal fragments (CTFs), soluble APP (sAPP), Aβ levels, and cleavage of an APP-GAL4 reporter protein. We report that direct stimulation of AMPAR increases non-amyloidogenic α-secretase-mediated APP processing and inhibits Aβ production.

## Materials and Methods

### Ethics Statement

Primary cortical neuronal cultures were prepared from mouse embryos as described previously [17] in accordance with UK Home Office Guidelines as stated in the Animals (Scientific Procedures) Act 1986 using procedures approved by the King's College London Ethics Committee.

### Antibodies

Rabbit polyclonal antibody CT20 raised against residues 676–695 of human APP (APP695 numbering) has previously been described [22]; mouse monoclonal APP antibody 13-M raised against a 21 amino acid sequence in the N-terminal domain of human APP, and which is identical in mouse and rat APP, was purchased from Alpha Diagnostic International (San Antonio, TX); phospho-ERK1/2 (Thr202/Tyr204) rabbit polyclonal antibody was purchased from Cell Signaling Technology (Danvers, MA); Glu1 (GluA1) polyclonal antibody, GluR2 (GluA2) polyclonal antibody and PSD-95 monoclonal antibody were purchased from Millipore (Temecula, CA); total ERK (C-14) rabbit polyclonal antibody was purchased from Santa Cruz Biotechnology (Santa Cruz, CA); synaptophysin (clone SVP-38) mouse monoclonal antibody was purchased from Sigma (St. Louis, MO); AlexaFluor 488 and AlexaFluor 546 secondary antibodies were purchased from Invitrogen (Carlsbad, CA); and horseradish peroxidase-conjugated secondary antibodies were purchased from Millipore (Temecula, CA). Unless otherwise indicated, primary antibodies were used for immunoblotting at the following dilutions: APP CT20 (1∶20,000); APP 13-M (1∶2,500); total ERK (1∶2,000); phospho-ERK1/2 (1∶2,500); synaptophysin (1∶2,000).

### Compounds

AMPA, NMDA, (+)-MK801, Nimodipine, DAPT, U0126, PD184352 and Fura2-AM were purchased from Tocris Bioscience (Bristol, UK); GYKI53655 was a gift from Lilly (Indianapolis, IN); EGTA was purchased from Sigma (St. Louis, MO); β-secretase inhibitor IV was purchased from Calbiochem (San Diego, CA); TAPI-1 was purchased from Peptides International (Louisville, KT); Complete Protease Inhibitor Cocktail tablets were purchased from Roche Applied Science (Indianapolis, IN).

### Plasmids

pRC-CMV vector containing human APP695 fused at the C-terminus via a 5 glycine hinge to the yeast transcription factor Gal4 containing both the DNA-binding and activation domains (APP695-Gal4) was originally provided by Professor Tommaso Russo (Naples, Italy), [23]. pFR-Luciferase reporter vector containing the firefly (*Photinus pyralis*) luciferase gene under the control of a synthetic promoter consisting of five tandem repeats of the yeast GAL4 activation sequence upstream of a minimal TATA box, and phRL-TK vector containing the Sea Pansy (*Renilla reniformis*) luciferase gene under the control of the herpes simplex virus thymidine kinase (HSV-TK) promoter, were from Promega (Madison, WI).

### Primary neuronal culture

Briefly, cortices were dissected from embryonic day 15 to 16 Swiss mouse embryos (NIH, Harlan, UK) and mechanically dissociated using a fire-polished glass Pasteur pipette in Hank's Buffered Salt Solution (Ca^2+^- and Mg^2+^-free). Neurons were plated into Nunc (Rochester, NY) multiwell tissue culture plates that had been coated previously with 20 µg/ml poly-D-lysine (Sigma, St. Louis, MO) and were maintained in Neurobasal medium without Phenol Red, supplemented with B-27, 2 mM glutamine, 100 µg/ml streptomycin, and 60 µg/ml penicillin (Invitrogen, Carlsbad, CA), at 37°C in a humidified atmosphere of 95% air and 5% CO_2_. Cultures used after 8–14 days in vitro (DIV), were 97–99% neuronal, as judged by β-tubulin III staining. Glial elements were typically less than 2%, as judged by GFAP staining.

### Immunofluorescence and image acquisition

Primary cortical neurons at 10 DIV, cultured on glass coverslips were fixed at room temperature in PBS (pH 7.4) containing 4% paraformaldehyde for 20 min. Neurons were then incubated in blocking-permeabilisation buffer (PBS, pH 7.4 containing 3% BSA, 0.1% TX-100) for 15 min. Primary antibodies: APP CT20 (1∶1,500 dilution), GluR1 (1∶500 dilution), GluR2 (1∶300), PSD-95 (1∶500 dilution), synaptophysin (1∶200 dilution) were applied in antibody buffer (PBS, pH 7.4 containing 1% BSA) for 18 h at 4°C, followed by application of AlexaFluor 488 (1∶1,000 dilution) or AlexaFluor 546 (1∶1,000 dilution) secondary antibodies in antibody buffer for 1 h followed by DAPI (600 nM) in PBS for 30 min. Immunofluorescence in the absence of primary antibodies produced a very weak, diffuse staining of cell bodies that did not overlap with the primary antibody-specific staining (data not shown). Multi-channel fluorescence (DAPI-FITC-Rhodamine filter set) images were captured using either a 40X or 63X oil objective fitted to a Zeiss META LSM510 confocal microscope, using Zeiss LSM image examiner software (Carl Zeiss, Thornwood, NY). Multi-channel image overlays were obtained using ImageJ software (NIH, USA).

### Single cell calcium imaging

Primary mouse cortical neurons grown on coverslips for 10 DIV were loaded with Fura-2 AM (5 µM; Invitrogen) at 37°C for 45 min. Dye loading and subsequent experiments were performed in HEPES-buffered saline (HBS; 140 mM NaCl, 5 mM KCl, 10 mM glucose, 10 mM HEPES, 2 mM CaCl_2_, and 1 mM MgCl_2_, pH 7.4) AMPA (50 µM) and antagonists where appropriate were applied to cells at room temperature by microperfusion. Images of individual cells typically 15–20 per field were captured every 2 s at 340 and 380 nm excitation wavelengths, with emission measured at 520 nm, using a microscope-based Concord imaging system. Analysis of emission intensity ratios at 340/380 nm was performed with the ImageMaster suite software (Photon Technology International, West Sussex, UK). Data was statistically analysed using GraphPad Prism software, Version 5 (La Jolla, CA).

### Detection of APP695 and APP C-terminal fragments (CTFs)

To detect APP695 and APP CTFs, primary cortical neurons cultured for 10 DIV were treated directly with agonists, antagonists and inhibitors as detailed in the legend to figures, washed once with PBS (pH 7.4), and lysed immediately in 100 µl SDS-PAGE sample buffer (62.5 mM Tris, pH 6.8, 2% SDS, 5% 2-mercaptoethanol, 10% glycerol and 0.0025% bromophenol blue), and then boiled for 5 min. For APP695 detection, samples were resolved by 8% Tris-glycine SDS-PAGE, and for APP CTFs detection, samples were resolved by 16.5% Tris-tricine SDS-PAGE (cathode buffer: 0.1 M Tris, 0.1 M tricine, 0.1% SDS; anode buffer: 0.2 M Tris-HCl, pH 8.9). After gel electrophoresis, proteins were transferred to 0.45 µm nitrocellulose (GE, Healthcare, Piscataway, NJ) for APP695 detection, or 0.2 µm Immobilon PVDF membranes (Millipore, Billerica, MA) for APP CTFs detection. Immunoblotting for APP695 was performed using primary polyclonal APP antibody CT20 (1∶20,000 dilution) and horseradish peroxidase-conjugated goat-anti-rabbit IgG secondary antibody (1∶20,000 dilution). Immunoblotting for APP-CTFs was performed using primary polyclonal APP antibodies CT20 (1∶75,000 dilution) and horseradish peroxidase-conjugated goat-anti-rabbit IgG secondary antibody (1∶200,000 dilution). APP695 bands were detected using the ECL system (GE Healthcare, Piscataway, NJ), and APP CTFs bands were detected using the ECL Advance system (GE Healthcare, Piscataway, NJ), followed by exposure to Hyperfilm ECL according to the manufacturer's instructions (GE Healthcare, Piscataway, NJ).

### Detection of Soluble APP (sAPP)

For detection of sAPP in the neuronal culture medium, primary neurons cultured for 10 DIV were treated as detailed in the legend to figures, followed by removal of the neuronal culture medium into tubes containing Complete Protease Inhibitor Cocktail. Samples were then centrifuged at 20,000× g for 15 min at 4°C to remove cell debris, and boiled for 5 min in SDS-PAGE sample buffer. Samples were resolved by 8% SDS-PAGE and transferred to 0.2 µm Immobilon PVDF membranes (Millipore, Billerica, MA). Immunoblotting was performed using primary monoclonal antibody APP 13-M (1∶2,500 dilution) and horseradish peroxidase-conjugated goat-anti-mouse IgG secondary antibody (1∶50,000 dilution), and immunoreactive bands were detected using the ECL Plus detection system and Hyperfilm ECL according to the manufacturer's instructions (GE Healthcare, Piscataway, NJ).

### Assessment of neuronal cell death

#### Lactate dehydrogenase (LDH) release

Primary cortical neurons cultured for 10 DIV were treated with AMPA (50 µM) for 20 min to 24 h. AMPA-induced cytotoxicity was evaluated by release of the cytosolic enzyme LDH into the culture medium using the CytoTox 96 non-radioactive cytotoxicity assay according to the manufacturer's instructions (Promega, Madison, WI), as described previously [24]. Absorbance was measured at 490 nm using a VersaMax microplate reader. Background LDH release (neuronal culture medium alone) was subtracted from the experimental values.

#### Phase contrast microscopy

Assessment of the effect of AMPA on neuronal morphology was made by phase contrast microscopy. Images were captured with a Zeiss AxioCam MRm cooled mono digital camera set at 1388X1040 pixels resolution and AxioVision (release 4.6) imaging software, using an Achrostigmat LD 32X0.4 NA Ph1 objective, fitted to a Zeiss Axiovert S100 microscope (Carl Zeiss, Thornwood, NY).

### Mouse Aβ_1–40_ ELISA

To determine the effect of AMPA receptor activity on Aβ_1–40_ release, conditioned medium from primary cortical neurons at 10 DIV was removed and neurons were washed twice with warm (37°C) PBS (pH 7.4) in order to remove Aβ that had accumulated with time in culture. Fresh warm (37°C) Neurobasal medium without Phenol Red, supplemented with B-27, containing vehicle or drug treatments, was added to the neurons for 6 h. Neurobasal medium was subsequently incubated with Complete Protease Inhibitor Cocktail and centrifuged at 100,000× g for 30 min at 4°C. Samples were then added to a mouse/rat Aβ_1–40_ ELISA plate (Immuno-Biological Laboratories (IBL), Code No. 27720), and processed for detection of Aβ_1–40_ according to the manufacturer's instructions (Immuno-Biological Laboratories (IBL), Minneapolis, MN). Mouse Aβ_1–40_ levels were calculated from a mouse/rat Aβ_1–40_ standard curve.

### Transfection of primary cortical neurons and Dual-Glo luciferase reporter gene activity assay for quantification of βγ -secretase-mediated cleavage of a human APP695-Gal4 fusion protein

pFR-Luciferase reporter plasmid (0.5 µg) was transfected in combination with an APP695-GAL4 plasmid (0.5 µg), into primary cortical neurons at 8 DIV using Lipofectamine 2000 (Invitrogen, Carlsbad, CA). All wells were co-transfected with phRL-TK plasmid (0.5 µg) that constitutively expresses moderate levels of *Renilla* luciferase. The total amount of DNA transfected into each well was 2 µg. Transfection mixes containing lipid and DNA were prepared in Opti-MEM I reduced serum medium (Invitrogen, Carlsbad, CA) by vortexing for 1 second and leaving for 25 min. Neuronal cultures were removed from the incubator and transfection mixes (150 µl per well) added dropwise onto the neuronal culture medium, after which, neuronal cultures were returned to the incubator. Neurons were treated with various compounds, as described processed 24 h after transfection for quantification of firefly luciferase reporter and constitutive Renilla luciferase expression as described previously [17]. Briefly, neurons were lysed with Glo Lysis Buffer (40 µl per well) (Promega, Madison, WI), and the Dual-Glo luciferase activity assay performed according to the manufacturer's instructions (Promega, Madison, WI). Luciferase signals were captured using a Veritas microplate luminometer (Turner BioSystems, Sunnyvale, CA). Firefly luciferase reporter activity was normalized using the constitutive *Renilla* luciferase activity, which helps to differentiate between specific and non-specific cellular responses and also controls for transfection efficiencies across experiments.

### Quantification and statistics

Immunoblot Hyperfilm ECL bands were quantified by scanning into ImageJ software at a resolution of 1200 dpi using an Epson Perfection V700 Photo flatbed scanner fitted with a Transparency Unit, and the mean background-corrected optical density (O.D.) of each band was interpolated from an O.D. calibration curve created using an O.D. step-tablet. Only Hyperfilm exposures that gave band O.D. values that were within the linear range of the O.D. calibration curve were used for statistical analysis. Meaned data ±SEM were graphed using GraphPad Prism software (La Jolla, CA). Immunoblot, Aβ_1–40_ ELISA and Dual-Glo luciferase activity assay data were analyzed by one-way ANOVA with either Dunnett's or Bonferroni post hoc tests, or by two-tailed Student's t tests, using GraphPad Instat software (La Jolla, CA). Differences between experimental treatments were considered to be statistically significant when p<0.05.

## Results

### Cortical neurons express postsynaptic AMPAR that are functionally coupled to ERK phosphorylation and APP cleavage

**Figure 1:**
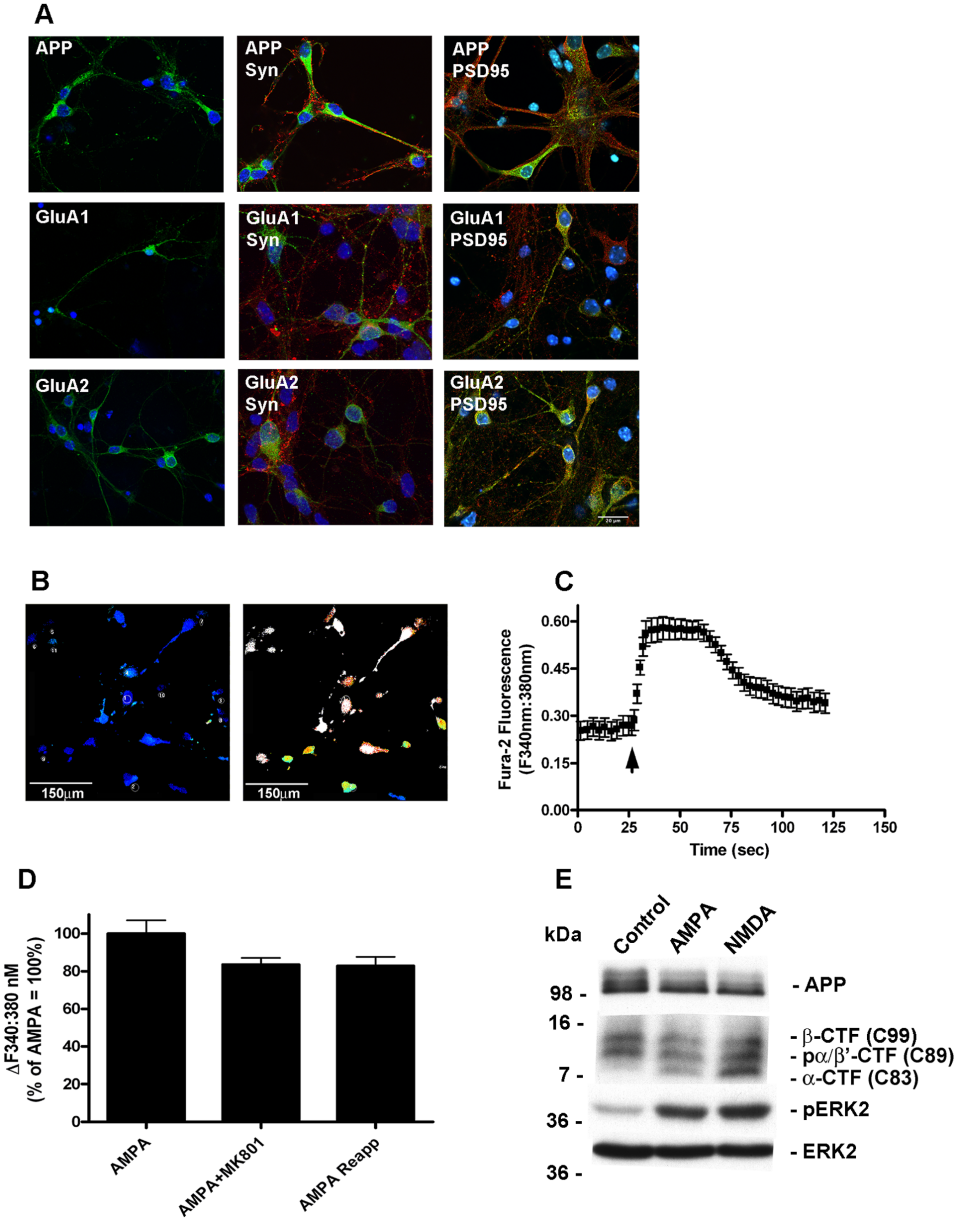
Cortical neurons express postsynaptic Ca^2+^-permeable AMPAR that are functionally coupled to ERK phosphorylation and APP cleavage. A, Immunofluorescence staining of primary cortical neurons at 10 DIV. Left hand panels; single immunofluorescence staining for APP, GluA1 and GluA2. Middle panels; double immunofluorescence staining for APP, GluA1 and GluA2 (all green) with the nerve terminal marker synaptophysin (Syn, red). Right hand panels; double immunofluorescence staining for APP, GluA1 and GluA2 (all green) with the postsynaptic marker PSD95 (red). Nuclei are counterstained with DAPI (blue), scale bar 20 µM. B, Fura-2 AM microfluorimetry demonstrating AMPA-evoked increases in Ca^2+^ in primary cortical neurons. Pseudocoloured images illustrating [Ca^2+^]i at baseline (left hand panel) and maximum [Ca^2+^]i response evoked by 50 µM AMPA (right hand panel), scale bar 150 µM. C, Representative ratiometric 340 nm∶380 nm trace against time (s) for Fura-2 AM loaded cortical neurons microperfused with 50 µM AMPA. D, Average changes in [Ca^2+^]i (340 nm∶380 nm ratio) of 40 individual neurons in response to sequential application of 50 µM AMPA, 50 µM AMPA+2.5 µM MK801 and reapplication of 50 µM AMPA (AMPA Reapp) expressed as % of the initial AMPA response. E, Primary cultured cortical neurons at 10 DIV were treated with vehicle (Control), 50 µM AMPA or 50 µM NMDA for 1 h followed by immunoblotting of neuronal lysates with antibodies to APP CT20 to detect full length APP695 (APP) and APP CTFs, phosphorylated ERK2 (pERK2), and ERK2.

**Figure 2:**
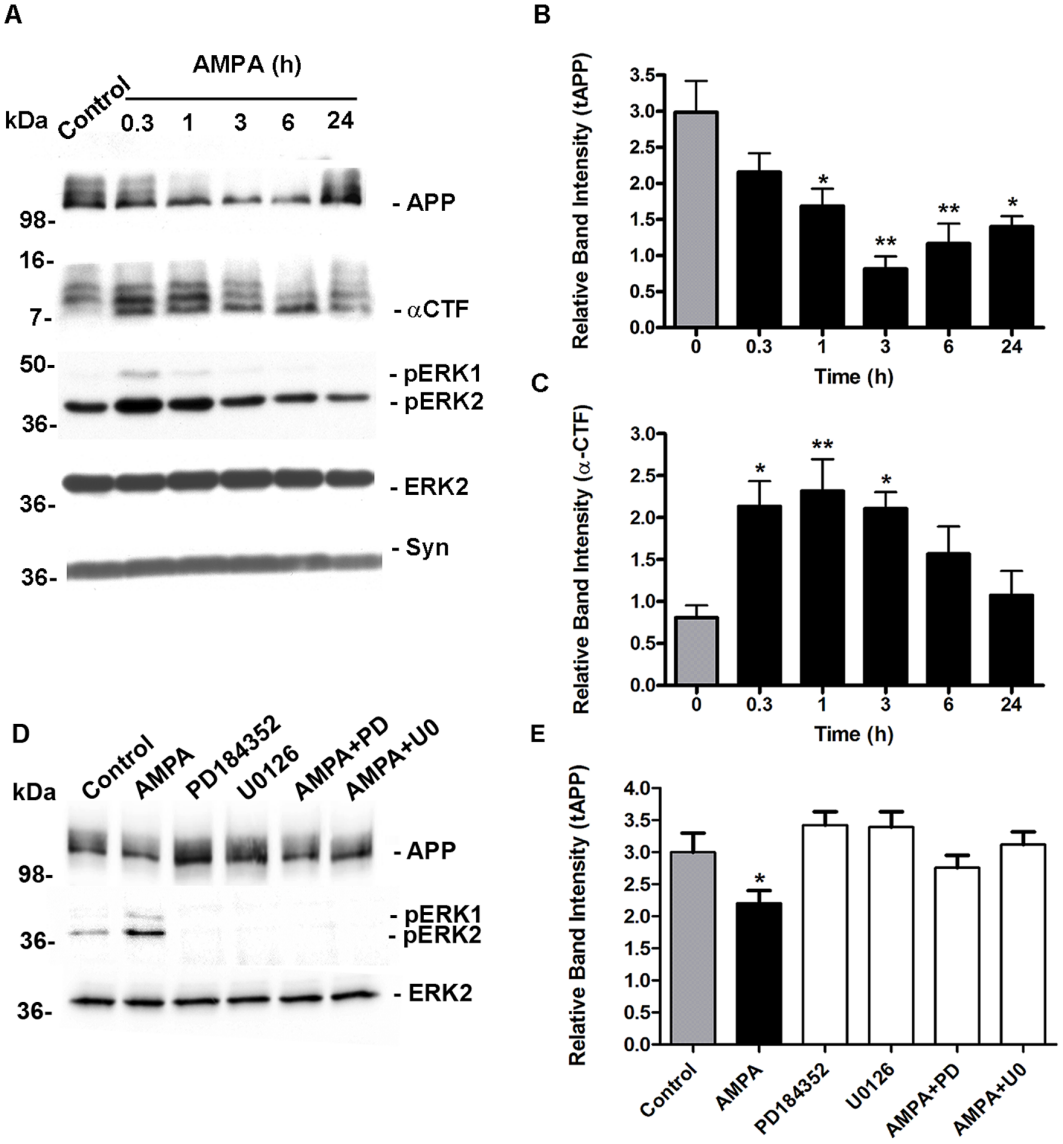
AMPAR-stimulation increases time-dependent APP processing that is not completely dependent on ERK activation. A, Primary cultured cortical neurons at 10 DIV were treated with vehicle (Control) or 50 µM AMPA for 0.3, 1, 3, 6 and 24 h followed by immunoblotting of neuronal lysates with antibodies to APP CT20 to detect full length APP695 (APP) and APP CTFs (α-CTF), phospho ERK1/ERK2 (pERK1/pERK2), ERK2 and synaptophysin (Syn). B, Full length APP695 levels (tAPP) and C, APP CTF (α-CTF) levels were analysed by ECL protein band densitometry using calibrated ImageJ software. Each column is the mean of +/−SEM of five independent experiments (n = 5; *p<0.05, **p<0.01, control (time 0, grey bar) vs treatment (black bars), one-way ANOVA with Dunnett's post hoc test). D, Primary cultured cortical neurons at 9 DIV were treated with vehicle (Control), 50 µM AMPA, 2 µM PD184352, 5 µM U0126 or AMPA in the presence of either PD184352 (AMPA+PD) or U0126 (AMPA+U0) for 1 h followed by immunoblotting of neuronal lysates with antibodies to APP CT20 to detect full length APP695 (APP), phospho ERK1/ERK2 (pERK1/pERK2) and ERK2. E, Full length APP695 levels (tAPP) were analysed by ECL protein band densitometry using calibrated ImageJ software. Each column is the mean of +/−SEM of five independent experiments (n = 4; *p<0.05, control (grey bar) vs AMPA (black bars), one-way ANOVA with Dunnett's post hoc test).

APP is sorted equally to the axonal and somatodendritic compartment in primary neurons [25] with extensive expression found postsynaptically [17], [26], [27]. We conducted an analysis of the distribution pattern of endogenous APP in cultured cortical neurons and similarly found widespread expression of APP, with some co-localisation with the nerve terminal marker synaptophysin and the postsynaptic marker PSD-95 (Fig 1A upper panels). We subsequently analysed the distribution of two AMPAR subunits, GluA1 and GluA2, and detected extensive co-localisation of these subunits with PSD-95 and staining around the cell soma but very little co-localisation with synaptophysin, suggesting that synaptic AMPAR are predominately postsynaptic in cultured cortical neurons and not located at nerve terminals (Fig 1A, middle and lower panels). Collectively, these data suggest that a pool of APP is expressed postsynaptically along with GluA1, GluA2 and PSD-95. The subunit composition of AMPA receptors determines their ion channel properties; receptors that possess the edited GluA2 subunit show low calcium permeability in comparison to unedited GluA2 receptors, which have high calcium permeability [28]. To determine the calcium permeability status of AMPAR in our cultures we examined AMPA-mediated increases in intracellular calcium using single cell Fura-2 AM microfluorimetry. Application of AMPA (50 µM) caused a rapid increase in intracellular calcium levels in the majority of neurons (Fig 1B, 1C) which was not blocked by the NMDAR open channel blocker MK801 (Fig 1D) or by L-type voltage sensitive calcium channel Nimodipine (not shown). This suggests that despite widespread expression of GluA2 the majority of cultured cortical neurons possess calcium permeable AMPAR, consistent with our previous findings [29]. AMPA stimulation of ERK phosphorylation was then investigated to confirm functional coupling of AMPAR to downstream physiological effectors. Treatment of neurons with AMPA (50 µM) for up to 1 h, caused a robust increase in ERK phosphorylation, to a similar level seen upon exposure to NMDA (50 µM) and with no detectable changes in the level of total ERK (Fig 1E). We next determined if stimulation of AMPAR led to the cleavage of full length of APP, which would be expected if AMPAR couple to APP processing. Cell lysates from cortical neurons treated with vehicle control, AMPA (50 µM) or NMDA (50 µM) for 1 h, were immunoblotted with APP antibody CT20 that is specific for the C-terminal residues 676–695 of APP695, and which will detect all full-length unprocessed cellular APP isoforms. We observed one discrete polypeptide band and two diffuse bands running just above the 98 kDa molecular weight marker (Fig 1E). Based on previous findings [17], [30] we assigned the lowest and major band as N-linked immature APP695, and the diffuse bands as the N- and O-linked mature forms of APP695. Upon stimulation with either AMPA or NMDA there was a reduction in the levels of the higher diffuse APP bands, indicative of processing of mature APP695. We also assessed the profile of APP-CTFs in the same cell lysates. We observed three discrete bands in the 7–16 kDa marker range, and based on our previous extensive characterisation [17] we concluded that the lowest molecular weight band was non-phosphorylated C83 generated from α-secretase activity (α-CTF). The next and noticeably stronger band was a mixture of phosphorylated C83 and β-secretase-generated non-phosphorylated C89, and the third band phosphorylated C89 and C99 generated from β-secretase cleavage (β-CTF). Treatment with AMPA, similarly to NMDA, increased α-secretase-generated C83 levels compared with control, consistent with enhanced α-secretase activity (Fig 1E). Having confirmed that AMPAR activation was able to modulate full length APP695 levels, a more detailed kinetic analysis was undertaken. Neurons were treated with AMPA (50 µM) for between 20 min to 24 h. Cells were lysed and samples immunoblotted for APP, pERK, total ERK and synaptophysin. Stimulation of AMPAR led to a time-dependent loss of mature APP, clearly detected at 20 min, reaching a maximum at 3 h and maintained for up to 24 h, although there was some indication of a partial recovery in APP levels at the longest time point (Fig 2A and 2B). In parallel to loss of full length APP, a time-dependent increase in the level of α-CTFs was also seen, consistent with enhanced α-secretase activity. The increase in α-CTFs was maintained above basal level for 3–6 h (Fig 2A, 2C) and at these longer time points there was a marked decrease in the level of the highest molecular weight CTF band. This is consistent with a reduction in the production of β-CTFs, and suggesting a reciprocal relationship between α- and β-secretase processing pathways following AMPAR activation. ERK phosphorylation followed the same kinetic as full length APP reduction and increased α-CTF generation, which peaked at 20 min and was still detectable at 1 h, and diminished thereafter, consistent with increased ERK phosphatase activity. Over 24 h treatment there was no change to either total ERK or synaptophysin levels and no detectable increase in LDH release (data not shown), suggesting no significant cell or synaptic damage was occurring during periods of prolonged AMPAR stimulation.

### AMPAR-evoked APP processing is only partially ERK-dependent

NMDAR-mediated non-amyloidogenic APP processing is dependent on the ERK pathway and AMPAR couple to this cascade via PI3-kinase [21]. To investigate whether AMPAR-evoked APP processing involved the ERK signaling pathway, cultured neurons were pre-treated with the MEK inhibitors PD184352 (2 µM) or U0126 (5 µM) for 5 min prior to bath application of AMPA (50 µM) for 1 h. Inhibition of MEK with either PD184352 or U0126 abolished basal and AMPAR-induced ERK phosphorylation as expected and caused a modest (∼15%) increase in APP levels (Fig 2D, 2E) suggesting a potential role for ERK in tonic constitutive APP processing. AMPAR-evoked loss of total APP was partially blocked by PD184352 and U0126, the extent of the reduction could be accounted for by the loss of constitutive APP processing in cultured neurons.

### AMPAR -mediated α-CTF production is independent of NMDARs and L-VSCC-dependent Ca^2+^-influx but requires ADAM metalloprotease activity

**Figure 3:**
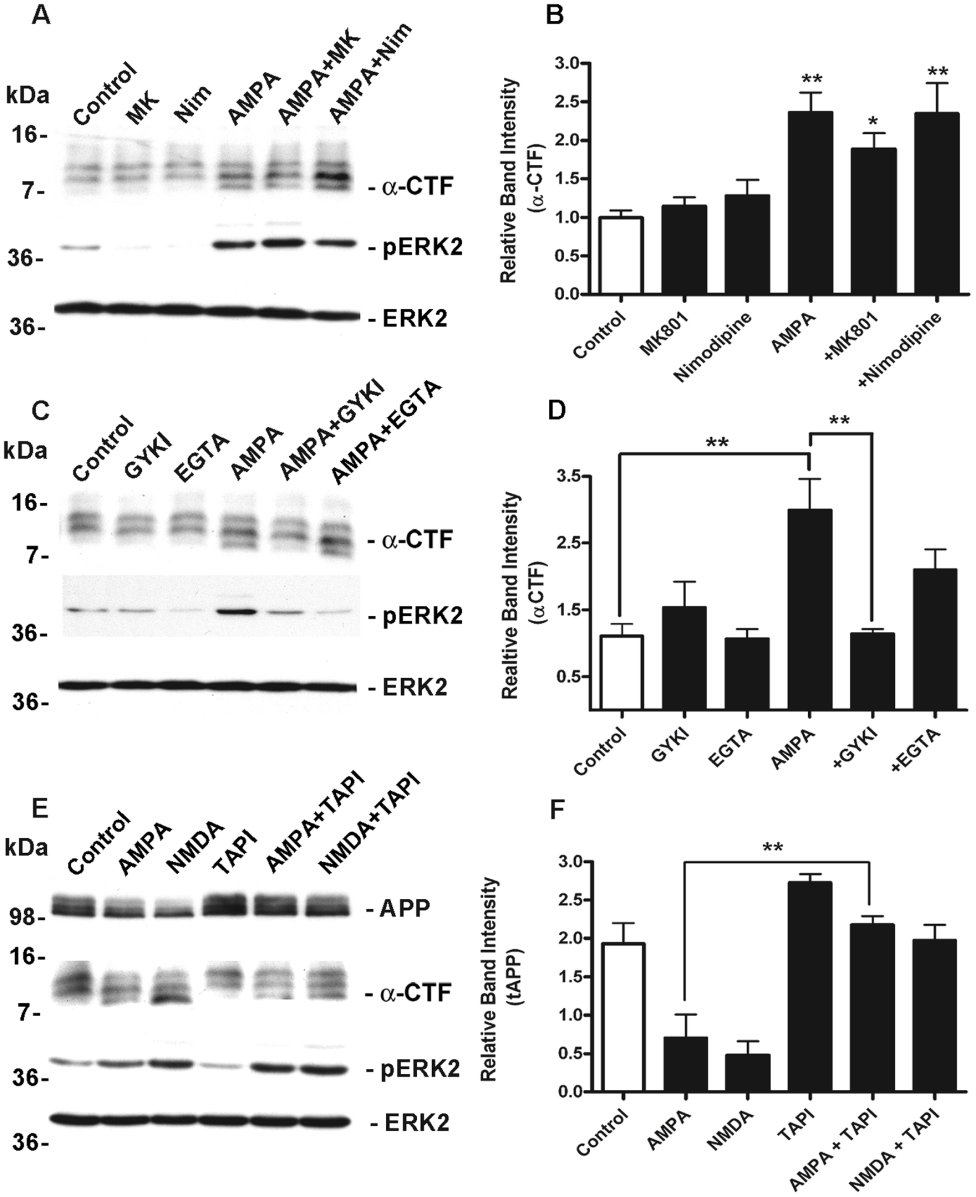
AMPAR-stimulation of APP processing is independent of NMDAR and L-type calcium channels is partially dependent on extracellular Ca^2+^ and is blocked by the ADAM inhibitor TAPI-1. A, Primary cultured cortical neurons at 10 DIV were treated for 1(Control), 50 µM AMPA, 2.5 µM MK801 (MK), 10 µM Nimodipine (Nim) or AMPA in the presence of either MK801 (AMPA+MK) or Nimodipine (AMPA+Nim) followed by immunoblotting of neuronal lysates with antibodies to APP CT20 to detect APP CTFs (α-CTF), phospho ERK1/ERK2 (pERK1/pERK2) and ERK2. B, APP CTF (α-CTF) levels were analysed by ECL protein band densitometry using calibrated ImageJ software. Each column is the mean of +/−SEM of six independent experiments (n = 6; *p<0.05, **p<0.01, control (white bar) vs AMPA, AMPA+MK801 and AMPA+Nimodipine (black bars), one-way ANOVA with Dunnett's post hoc test). C, Primary cultured cortical neurons at 10 DIV were treated for 1 h with vehicle (Control), 50 µM AMPA, 10 µM GYKI53655 (GYKI), 2 mM EGTA or AMPA in the presence of either GYKI53655 (AMPA+GYKI) or EGTA (AMPA+EGTA) followed by immunoblotting of neuronal lysates with antibodies to APP CT20 to detect APP CTFs (α-CTF), phospho ERK1/ERK2 (pERK1/pERK2) or ERK2. D, APP CTF (α-CTF) levels were analysed by ECL protein band densitometry using calibrated ImageJ software. Each column is the mean of +/−SEM of five independent experiments (n = 5; **p<0.01, control (white bar) vs AMPA (black bar) and AMPA vs AMPA+GYKI (black bars) one-way ANOVA with Bonferroni post hoc test). E, Primary cultured cortical neurons at 10 DIV were treated for 1 h with vehicle (Control), 50 µM AMPA or 50 µM NMDA, in the absence and presence of 50 µM TAPI-1 (AMPA+TAPI, NMDA+TAPI) followed by immunoblotting of neuronal lysates with antibodies to APP CT20 to detect APP CTFs (α-CTF), phospho ERK1/ERK2 (pERK1/pERK2) or ERK2. F, APP CTF (α-CTF) levels were analysed by ECL protein band densitometry using calibrated ImageJ software. Each column is the mean of +/−SEM of five independent experiments (n = 5; **p<0.01, AMPA vs and AMPA+TAPI (black bars) one-way ANOVA with Bonferroni post hoc test).

Although the majority of neurons in our cultures appeared to express Ca^2+^-permeable AMPAR determining whether AMPA-evoked ERK phosphorylation and APP processing was direct or secondary to membrane depolarization and activation of NMDAR or L-VSCCs was next investigated. Cultured neurons were therefore pre-treated with the non-competitive NMDA receptor antagonist MK801 (2.5 µM) or the L-VSCC blocker Nimodipine (10 µM), prior to bath application of AMPA (50 µM) for 1 h. AMPAR activation induced a significant increase in α-CTF generation and ERK phosphorylation, which were not inhibited by either MK801 or Nimodipine (Fig 3A and 3B). These data suggest the AMPA-mediated signaling and regulated APP metabolism are direct, and independent of the subsequent activation of either NMDA receptors or L-VSCCs following membrane depolarisation. Our previous investigations strongly implicated Ca^2+^ influx through NMDA receptors as a requirement for non-amyloidogenic processing of APP [17]. However, since secondary sources of Ca^2+^ influx through either NMDA receptors or L-VSCCs did not appear to contribute to AMPA-mediated APP metabolism, we set out to determine the extent to which the AMPA response depended upon extracellular Ca^2+^. Neurons were pre-treated with the selective non-competitive AMPAR antagonist GYKI53655 (50 µM), to confirm the direct involvement of AMPAR, or with the Ca^2+^ chelator EGTA (2 mM) to address the requirement for extracellular Ca^2+^, prior to bath application of AMPA (50 µM) for 1 h. As expected AMPAR activation resulted in a significant increase in ERK phosphorylation which was abolished by GYKI53655 and by chelation of extracellular Ca^2+^. These data confirm that in cultured neurons, ERK phosphorylation is mediated by influx of extracellular Ca^2+^ through AMPAR [21]. Similarly, treatment with AMPA resulted in a significant increase in α-CTF levels which was also abolished by GYKI53655. However, in contrast to ERK phosphorylation AMPA-mediated increases in α-CTFs were not completely blocked by EGTA suggesting that α-CTF production was only partially dependent on extracellular Ca^2+^ (Figure 3C and 3D). We previously demonstrated that NMDA-stimulated non-amyloidogenic metabolism of APP required an ADAM metalloprotease activity [17], most likely ADAM10 [18], [19]. As activity at AMPAR also appeared capable of promoting non-amyloidogenic APP metabolism to enhance the level of αCTFs, we sought to determine if these effects were also mediated by ADAM metalloprotease activity. Cultured neurons were pre-treated with the broad spectrum ADAM inhibitor TAPI-1 (50 µM) and stimulated with AMPA (50 µM) or NMDA (50 µM) for 1 h. Stimulation of AMPAR and NMDAR resulted in a robust increase in α-CTF levels with a concurrent reduction in full length APP and both of these effects were strongly inhibited by TAPI-1. AMPA and NMDA-induced ERK phosphorylation were unaffected by TAPI-1 and there were no changes in total ERK levels (Fig 3E and 3F). Collectively, these data provide strong evidence to support the involvement of ADAM metalloprotease activity in α-secretase-mediated cleavage of APP following AMPAR stimulation.

### AMPAR stimulation enhances sAPP release, inhibits βγ-secretase activity and reduces Aβ secretion

**Figure 4:**
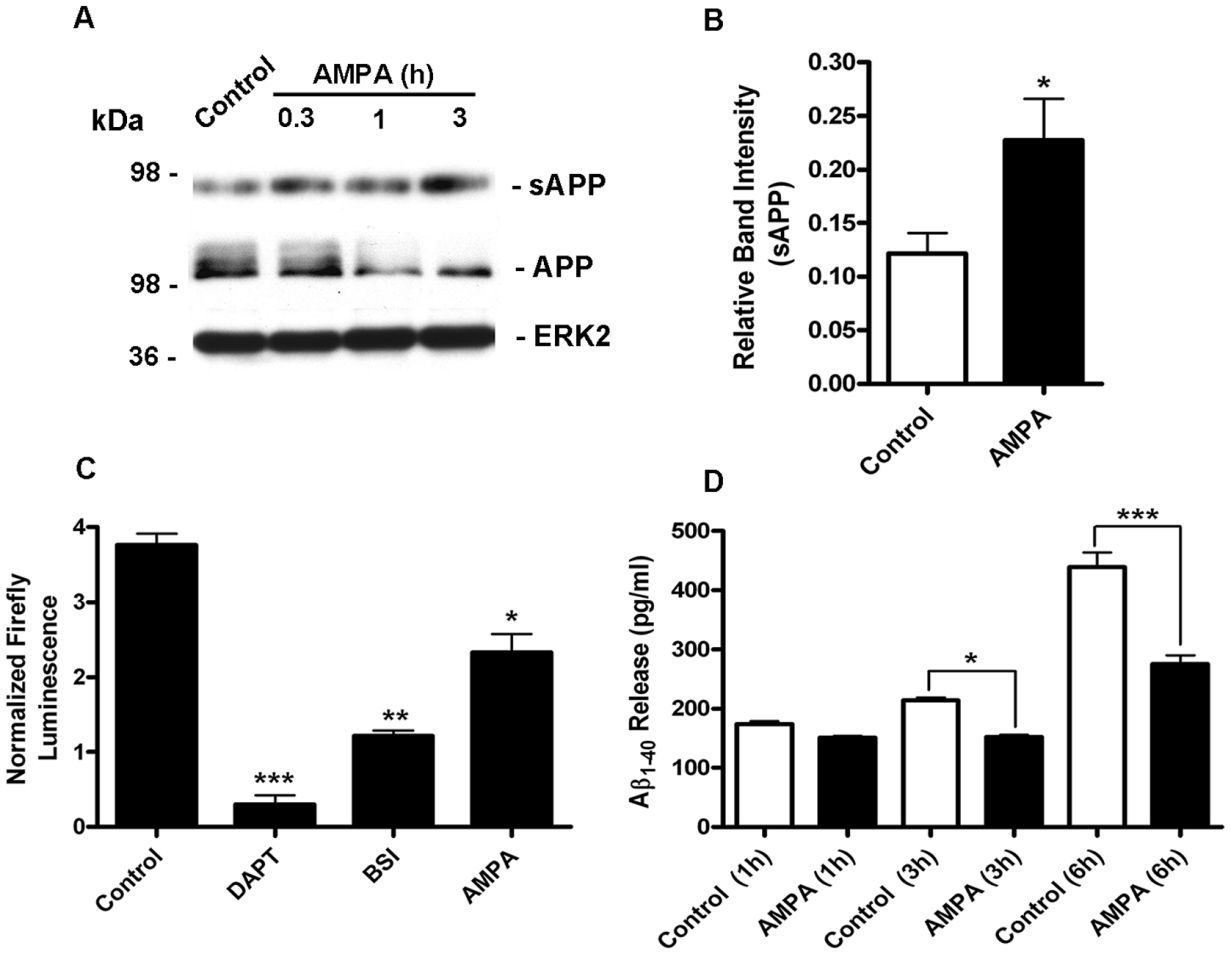
AMPAR-activity stimulates sAPP release, inhibits β-secretase processing and reduces Aβ secretion from primary cultured cortical neurons. A, Primary cultured cortical neurons at 10 DIV were treated with vehicle (Control) or 50 µM AMPA for 0.3, 1 or 3 h, followed by immunoblotting of the growth media with an N-terminal APP antibody APP13M to detect secreted APP (sAPP) and immunoblotting of the corresponding neuronal lysates with APP CT20 and ERK2. B, sAPP levels in the media from vehicle (Control) and following treatment with AMPA for 3 h were analysed by ECL protein band densitometry using calibrated ImageJ software. Each column is the mean +/−SEM of four independent experiments (n = 4; **p<0.05, Control (white bar) vs AMPA (black bar) unpaired two-tailed Student's t-test). C, Primary cultured cortical neurons at 8 DIV were cotransfected with APP695-GAL4, pFR-Luc Firefly luciferase reporter gene and phRL-TK plasmids and then treated with vehicle (Control), 10 µM DAPT, 10 µM β-secretase inhibitor (BSI) or 50 µM AMPA. Dual-Glo luciferase activity assays were performed 24 h after transfection for quantification of Firefly and Renilla luciferase expression. Firefly luciferase reporter activity was normalized using the constitutive Renilla activity. Each column is the mean +/−SEM of 12 separate transfections prepared from 3 independent cultures (n = 12; *p<0.05; **p<0.01; ***p<0,001; control vs DAPT, control vs BSI, control vs AMPA one-way ANOVA with Dunnett's post hoc test). D, Primary cultured cortical neurons at 10 DIV had a media change and were then treated with vehicle (Control) or 50 µM AMPA for 1, 3 or 6 h. The neuronal culture medium was removed and Aβ_1–40_ levels were measured by ELISA. Each column represents the mean +/−SEM of three independent experiments (n = 3; *p<0.05; ***p<0.001; control (white bars) vs AMPA (black bars).

During non-amyloidogenic processing, APP is cleaved by α-secretase to produce a membrane embedded α-CTF and releases a large soluble sAPPα ectodomain fragment from the cell surface. This ectodomain fragment is secreted into the extracellular space, decreases Aβ generation by directly associating with BACE-1 [31] and may have neuroprotective and memory enhancing properties [32]–[34]. It is thought that most amyloidogenic APP metabolism occurs intracellularly [35]. Therefore, levels of sAPP in the culture medium can be used as a general marker of non-amyloidogenic APP processing. Our observation that AMPA increased α-CTF levels, suggested that activation of AMPAR should also increase sAPP release. We stimulated cortical neurons with AMPA (50 µM) for up to 3 h and assessed subsequent sAPP release. Samples of conditioned medium were immunoblotted with an APP antibody (APP13-M) raised against a 21 amino acid sequence in the N-terminal ectodomain of human APP that also detects mouse, and rat sAPP. APP13-M detected a single band migrating just below the 98 kDa molecular weight marker compared with uncleaved full-length APP695 which runs above the 98 kDa molecular weight marker. No full-length APP immunoreactivity was detected in our neuronal medium samples, as judged by immunoblotting with a C-terminally directed APPCT20, which detects APP695 but not sAPP (data not shown). Stimulation of AMPAR produced a time-dependent increase in the levels of sAPP release concurrent with a decrease of mature full length cellular APP. Following 3 h of AMPAR activation the levels of sAPP in the medium were approximately double those detected at baseline which taken with the observed increase in α-CTF production and the overall sensitivity to TAPI-1 strongly suggests that stimulation of AMPAR promotes non-amyloidogenic APP processing (Figure 4A and 4B). Furthermore, we also tested the ability of AMPA to influence an AICD-Gal4-driven luciferase reporter which we have previously shown preferentially reports βγ-secretase-mediated APP processing in primary cortical neurons [17], [36]. The assay utilizes a reporter protein consisting of human APP695 fused at the C-terminus to the yeast transcription factor Gal4 containing both the DNA-binding and activation domains (APP695-Gal4). Upon normal proteolytic processing of APP695-Gal4 by γ-secretase an AICD-Gal4 fragment is formed that induces transcription through a UAS-luciferase reporter gene (pFR-Luc) by virtue of its transactivation domain and specific binding to a Gal4-UAS promoter via its DNA-binding domain. In order to confirm sensitivity to βγ-secretase processing cortical neurons were treated with the γ-secretase inhibitor DAPT or a small molecule BACE-1 inhibitor (Figure 4C). Consistent with our previous report [17], DAPT reduced luciferase activity by 85% and the BACE-1 inhibitor by 65%. Conversely, the α-secretase (ADAM) inhibitor TAPI-1 did not inhibit luciferase activity (data not shown). This confirms that in primary cultured neurons the β-secretase cleavage pathway of APP preferentially mediates AICD nuclear signalling and that this assay system preferentially monitors amyloidogenic (Aβ-forming) APP processing by β- and γ-secretase [37]. We found that treatment of primary cortical neurons with AMPA (50 µM) for 6 h, decreased the total luciferase activity by 40% compared with control activity (Figure 4C). AMPA had no significant effect on basal luciferase activity arising from the firefly luciferase reporter alone nor did it significantly alter the constitutive *Renilla* luciferase activity that was used to normalize firefly luciferase activity (not shown). Collectively this data strongly suggested that AMPAR activity inhibits β/γ-secretase-mediated APP processing. If this conclusion were correct then it followed that AMPAR stimulation should also reduce Aβ formation. To address this we investigated Aβ_1–40_ release in cultured cortical neurons using a rodent specific ELISA. This was carried out by replacing conditioned medium with fresh treatment media containing either vehicle or AMPA (50 µM) for 1, 3 or 6 h. Conditioned medium was replaced as Aβ accumulates in the medium with time, such that the extracellular levels become too high to be quantified by high sensitivity ELISA. In the absence of AMPA, Aβ_1–40_ release was cumulative such that at 6 h the levels of Aβ_1–40_ were more than double the values measured at 1 h. Aβ_1–42_ levels were below the detection limit (data not shown). In the presence of AMPA, there was a trend towards reduced levels of Aβ_1–40_ as early as 1 h and a significant reduction by 3 h when compared to control (Figure 4D). At 6 h, control levels of Aβ_1–40_ were substantially increased while Aβ_1–40_ levels in the AMPA (6 h) treatment group were not significantly different from control (1 h) levels, suggesting that AMPAR activation effectively reduces amyloidogenic metabolism over a wide range of time points such that there is almost no increase in Aβ levels within this time frame.

## Discussion

AMPAR have long been considered as potential therapeutic targets for neurological diseases associated with aberrant excitatory neurotransmission and excitotoxicity [38]. With respect to AD, this has focused mainly on assessing the potential use of AMPAR modulators to boost cognitive performance with rather disappointing results to date [39]. However, AMPAR may also be a potentially important locus for the actions of Aβ within the synapse since Aβ interacts with and internalizes AMPAR [40]–[43], and disrupts GluA trafficking to the cell surface [44]. These interactions could then lead to deficits in AMPAR-evoked transmission [45] and loss of the molecular events that are central to synaptic plasticity and survival, similar to the aberrant Ca^2+^ signaling, Akt and CREB phosphorylation that results from Aβ acting at NMDAR [46]–[48]. In contrast to AMPAR mediating the actions of Aβ, much less consideration has been given to the role that AMPAR might play in the development and progression of AD pathology by directly regulating APP metabolism, sAPP secretion and Aβ production. Here we report that activation of endogenous AMPAR in primary cultured cortical neurons enhances non-amyloidogenic APP processing to significantly increase α-secretase-generated αCTF and sAPP levels, and decrease Aβ release. The mechanism is independent of NMDAR and L-VSCCs, and is partially dependent on extracellular Ca^2+^

To provide a dynamic readout of α- and β-secretase-mediated proteolytic processing of APP we first analysed APP CTF production. We found that bath application of AMPA to cortical neurons for 20 min caused a strong upregulation in the levels of α-CTFs with no increase in β-CTFs, consistent with promotion of the α-secretase pathway. The increase in α-CTF levels was maintained for at least 6 h after application of AMPA, during which time there was a marked loss of β-CTFs suggesting a reciprocal relationship between the α- and β-secretase pathways following AMPAR stimulation which is consistent with the hypothesis that α- and β-secretase compete for APP as a substrate [49]–[50]. AMPA-evoked increase in the α-CTFs was blocked by the non-competitive AMPAR antagonist GYKI53655 but was unaffected by the NMDA receptor channel blocker MK801 or by the L-type VSCC blocker Nimodipine, suggesting direct coupling from AMPAR to APP cleavage. The increase in α-CTFs was however, only partially dependent on extracellular calcium despite AMPA-evoked ERK phosphorylation being abolished by EGTA. This is in contrast with our previous findings which showed that NMDAR stimulation of α-CTFs were entirely dependent on Ca^2+^ influx [17]. Possible explanations for the apparent Ca^2+^-independent component of APP processing is a mechanism relating to the known metabotropic signalling actions of AMPA receptors, independent of ion influx [20],[51] or signaling evoked from intracellular Ca^2+^ stores but this has not been explored.

The MEK/ERK pathway is known to regulate signal-responsive APP processing [52], [53] and NMDAR coupling to non-amyloidogenic processing has been strongly linked to activation of ERK [15], [19]. We found that APP levels were modestly increased in the presence of MEK inhibitors suggesting that basal turnover or processing of APP is ERK sensitive. NMDAR antagonists and channel blockers inhibit the α-secretase component of basal APP processing in cultured cortical neurons [17] and so this effect of MEK inhibitors alone is likely to be due to inhibition of endogenously released glutamate acting through NMDAR to regulate APP processing via the ERK pathway. Although AMPAR stimulation robustly activated ERK phosphorylation we could find no clear evidence that AMPAR activation of APP processing was strongly dependent on this pathway and the reduction in processing seen in the presence of MEK inhibitors was probably due to inhibition of the basal activity rather than effects on the AMPA-responsive component directly.

AMPAR-evoked increase in α-CTFs was blocked by TAPI-1 which is a general ADAM inhibitor acting at a range of ADAMs including ADAM10 and ADAM17 which are the most likely candidate signal-responsive α-secretases in neurons [6], [54], [55]. Whether AMPAR receptors recruit ADAMs to the cell surface via interactions with SAP97 as described for NMDAR [18] is not yet clear, but AMPAR subunit trafficking is mediated by SAP97 [56] so this is possible. Our findings that AMPAR activity increased α-CTF levels suggested to us that AMPAR receptor activation should also increase sAPPα release and in support of this we detected around a one and a half fold increase in the levels of sAPP in the medium after treatment with AMPA. To determine whether this increase in non-amyloidogenic APP cleavage was due to a shift from β- to α-secretase-mediated APP processing, we utilised an APP-GAL4 cleavage assay. β-secretase expression and activity is high in embryonic neurons and the APP-GAL4 assay preferentially reports βγ-secretase processing in this cell type [17] thus factors which increase the activity of α-secretase would be predicted to reduce UAS-driven luciferase expression. Indeed, AMPA caused a ∼40% reduction in luciferase expression consistent with a potentiation of α-secretase activity and a parallel reduction in β-secretase activity. We further reasoned that a sustained reduction in β-secretase-mediated processing should lower the levels of Aβ and to address this we employed an ELISA that detects rodent Aβ_1–40_. Background levels of Aβ are high in cultured neurons due to the strong preference for β-secretase processing and so ELISA detection of secreted Aβ needed to be conducted after a media change. Following the addition of fresh medium there was a time-dependent increase in the levels of Aβ_1–40_ which was very strongly inhibited by AMPAR stimulation suggesting that AMPAR can promote α-secretase processing and in doing so reduce β-secretase-mediated generation of Aβ although it is not yet clear to what extent this occurs *in vivo*. Somewhat in contrast to our findings here, infusion of AMPAR antagonists into mouse hippocampus suppresses ISF Aβ levels suggesting baseline glutamate signaling through AMPAR increases Aβ [15]. However, the effects of direct stimulation of AMPAR with agonists or potentiators was not reported so similar to NMDAR a strong stimulus could perhaps reduce Aβ production. Regulation of APP processing following the release of endogenous glutamate appears to favour a synaptic NMDAR route [17] but at AMPAR-enriched potentiated synapses a more direct pathway to regulate sAPP and Aβ levels might occur and in doing so exert additional control over excitability. In summary, we have shown that AMPAR can be added to the repertoire of receptors that couple to non-amyloidogenic APP processing at glutamatergic synapses and our results suggest that direct pharmacological targeting with AMPAR modulators or indeed with interventions that regulate GluA expression and signaling [57], [58] could potentially be exploited to slow the development and progression of Aβ pathology.
